# Risk Factors for the Mortality of* Pneumocystis jirovecii* Pneumonia in Non-HIV Patients Who Required Mechanical Ventilation: A Retrospective Case Series Study

**DOI:** 10.1155/2017/7452604

**Published:** 2017-05-08

**Authors:** Toru Kotani, Shinshu Katayama, Yuya Miyazaki, Satoshi Fukuda, Yoko Sato, Koichi Ohsugi

**Affiliations:** Department of Anesthesiology and Intensive Care Medicine, Tokyo Women's Medical University, Tokyo 162-8666, Japan

## Abstract

**Background:**

The risk factors for the mortality rate of* Pneumocystis jirovecii* pneumonia (PCP) who required mechanical ventilation (MV) remained unknown.

**Methods:**

A retrospective chart review was performed of all PCP patients admitted to our intensive care unit and treated for acute hypoxemic respiratory failure to assess the risk factors for the high mortality.

**Results:**

Twenty patients without human immunodeficiency virus infection required mechanical ventilation; 19 received noninvasive ventilation; and 11 were intubated. PEEP was incrementally increased and titrated to maintain FIO_2_ as low as possible. No mandatory ventilation was used. Sixteen patients (80%) survived. Pneumothorax developed in one patient with rheumatoid arthritis (RA). Median PEEP level in the first 5 days was 10.0 cmH_2_O and not associated with death. Multivariate analysis showed the association of incidence of interstitial lung disease and increase in serum KL-6 with 90-day mortality.

**Conclusions:**

We found MV strategies to prevent pneumothorax including liberal use of noninvasive ventilation, and PEEP titration and disuse of mandatory ventilation may improve mortality in this setting. Underlying disease of interstitial lung disease was a risk factor and KL-6 may be a useful predictor associated with mortality in patients with RA. These findings will need to be validated in larger studies.

## 1. Introduction

The incidence of* Pneumocystis jirovecii* pneumonia (PCP), a disease commonly occurring in patients with acquired immunodeficiency syndrome, has been increasing in patients without human immunodeficiency virus (HIV) infection as immunosuppressive medications become more widely used. Mortality of PCP depends upon the underlying disease and has been increasing [1], being 10% in HIV-positive and 30–60% in HIV-negative disease [2, 3]. Although the reasons for these differences in mortality rates are not yet fully understood, neutrophil counts in bronchoalveolar lavage fluid are significantly higher in HIV-negative than HIV-positive patients with PCP [4]. The symptoms of PCP are more abrupt and the incidence of tracheal intubation was 10-fold higher in HIV-negative than in HIV-positive patients [5], suggesting that more intense inflammatory responses develop in HIV-negative patients [2, 5].

In contrast, the overall mortality rate of PCP patients who required mechanical ventilation (MV) was reported to be 50% to 60% regardless of HIV infection [5]. In HIV-negative patients, increased mortality rate was associated with positive end-expiratory pressure (PEEP) on the first three days of acute respiratory failure [6] and the development of pneumothorax during MV [7, 8]. MV was a risk factor for the poor prognosis of patients with severe PCP [1, 2, 5, 7–9], but whether or not MV is simply a marker of severity or itself is harmful in PCP patients is unclear. The finding, that noninvasive positive pressure ventilation (NPPV) decreased the incidence of pneumothorax [10, 11], indicates that invasive ventilation may be causing harm. However, the association of the modalities of MV during invasive and noninvasive ventilation with the incidence of pneumothorax has never been investigated. Because two-thirds of patients presenting with acute respiratory failure required MV [12], the role of MV as a therapeutic modality should be investigated further.

We therefore retrospectively evaluated the effects of MV on 90-day mortality in HIV-negative patients with acute respiratory failure caused by PCP in our hospital. We sought the underlying conditions, clinical and laboratory features, ventilator settings, the incidence of tracheal intubation, and the adverse events during MV to assess the risk factors associated with poor patient prognosis.

## 2. Methods

### 2.1. Subjects

A retrospective chart review was performed of all patients admitted to our multidisciplinary general intensive care unit (ICU) from December 2008 until January 2012 who were diagnosed with PCP and treated for acute hypoxemic respiratory failure with any types of MV.

### 2.2. Diagnosis of PCP

Patients were suspected of PCP infection if they had a history of immunosuppressant use and manifested acute-onset dyspnea and hypoxemia at admission and if chest radiographs showed bilateral opacities. PCP was diagnosed by fluorescent antibody staining (Fungi-Fluor Kit Pneumocystis Kit, Polysciences Inc.) using induced sputum or bronchoalveolar lavage fluid. Results were checked by a trained laboratory technician and confirmed by the board-certified infection control doctors.

### 2.3. Treatment for PCP

#### 2.3.1. Medication

Intravenous administration of a combination of trimethoprim (15 mg/kg) and sulfamethoxazole (75 mg/kg) plus methylprednisolone was started immediately after the diagnosis of PCP. The dose of methylprednisolone was dependent on the prior dosage of steroids and any underlying diseases.

#### 2.3.2. Mechanical Ventilation

Because a specialized protocol of MV for PCP has not been developed, we used the MV protocol for acute hypoxemic respiratory failure without hypercapnia in our ICU. Briefly, NPPV was initiated with 7 cmH_2_O of continuous positive airway pressure (CPAP) and 1.0 of FIO_2_ via facemask using NPPV machine (BiPAP VISION, Fuji Respironics, Tokyo) when supplemental oxygen failed to maintain percutaneous oxygen saturation (SpO_2_) over 90%. Biphasic positive airway pressure (BIPAP) and pressure support ventilation (PSV) were not used during noninvasive ventilation. When NPPV was contraindicated, 12 cmH_2_O of CPAP failed to improve hypoxemia, or delayed intubation may put the patient at risk for adverse outcome as expected by an attending physician, MV was administered via tracheal intubation using an ICU-type ventilator (Evita-XL, Draeger Medical, Germany). CPAP or airway pressure release ventilation (APRV) with automatic tube compensation was used depending on the gas exchange. PEEP was titrated to maintain FIO_2_ as low as possible. CPAP was converted to APRV when PEEP of 18 cmH_2_O did not maintain PaO_2_ > 60 mmHg or when respiratory acidosis was observed. APRV was applied according to the previous review [13]. Briefly, PEEP (*P*_high_) was titrated to maintain acceptable oxygenation. The *T*_high_ was set to prevent hypercapnia or respiratory acidosis. *P*_low_ was zero and *T*_low_ was set to achieve ratios of termination of peak expiratory flow rate to peak expiratory flow rate of 50 to 75%.

#### 2.3.3. Data Collection

The following data were collected: general demographic information; underlying diseases including HIV status; underlying immunosuppressive conditions; time from the onset of pneumonia to the start of PCP treatment; medications used to treat PCP; laboratory analysis, including serum 1,3-*β*-D-glucan, lactate dehydrogenase (LDH), KL-6, and surfactant protein (SP)-D; acute physiological and chronic health evaluation (APACHE) II score at ICU admission; MV parameters; incidence of tracheal intubation; duration of MV, ICU stay, and hospital stay; and overall hospital mortality. Serum 1,3-*β*-D-glucan was measured using FangitecG MK (Seikagaku Corp.), with a cutoff of 20 pg/mL, a value set by our institution's laboratory department.

#### 2.3.4. Statistical Analysis

Statistical analyses were performed using JMP 9 (SAS Institute Inc., Cary, NC, USA). Proportion was used as descriptive statistics for categorical variables and mean ± SD or median (interquartile range (IQR)) for quantitative variables. Between-group differences were compared using Fisher's exact test for qualitative variables. A *t*-test or Mann–Whitney *U* test was used to compare quantitative variables, as appropriate. All variables that had a *p* value below 0.2 in the univariate analysis were entered into a stepwise multivariate analysis. All tests were two-tailed. Statistical significance was defined as *p* < 0.05.

## 3. Results

### 3.1. Demographic Data

During the study period, we suspected that 28 patients had PCP, based on their symptoms, radiographs, and history of underlying disease. Of these, 21 patients were diagnosed with PCP, and 20 (95%) required MV.

Demographic data of all patients are shown in Table 1. Nine patients had rheumatoid arthritis (RA) or other collagen vascular diseases, and seven were renal transplant recipients. Twelve patients (60%) were female, including all nine with RA. None of the study subjects was HIV-positive. Although all the patients had received chemoprophylaxis for PCP when the immunosuppression was initially started, the regimen of immunosuppressants was changed or stopped at some stages in each patient. No patients included in the study were given any chemoprophylaxis just before the onset of PCP. Patients with RA/collagen disease received 7.0 (2.9, 10.0) mg methotrexate per week. None of these patients received any biologic agents within one year before PCP infection. Basic immunosuppressants in renal transplant recipients included mycophenolate mofetil and tacrolimus or cyclosporin. Prednisolone-equivalent dose in patients with RA/collagen disease and renal transplants was 3.0 (1.3, 7.8) and 5.0 (5.0, 8.2) mg/day, respectively. Treatment started 3.5 (1.0, 7.0) days after admission.

### 3.2. MV Parameters and Duration

NPPV was used in 19 patients. Eleven patients (55%) were switched to tracheal intubation because of the failure of NPPV (7 cases), sustained NPPV (3), or the development of pneumothorax (1). One patient could not tolerate the facemask and was intubated without NPPV. PEEP level at days 1, 3, and 5 was 10.0 (8.0, 10.5), 10.0 (8.0, 14.3), and 10.0 (8.0, 14.0) cmH_2_O, respectively. Durations of NPPV, tracheal intubation, and MV were 3.5 (1.0, 9.3), 5.0 (0.0, 12.3), and 10.0 (5.0, 15.5) days, respectively. The longest duration of MV including NPPV was 71 days in a patient who survived. Eight patients (40%) did not require the intubation; their CPAP level on day 1 was 7.5 (6.8, 8.0) cmH_2_O and their duration of NPPV was 5.5 (3.8, 9.3) days.

### 3.3. Serum Biomarkers at ICU Admission

Serum 1,3-*β*-D-glucan, LDH, KL-6, and SP-D at ICU admission were presented in Table 2. Data in total and two major subgroups, RA/collagen disease patients and renal transplantation recipients, were listed. Serum 1,3-*β*-D-glucan concentration was significantly lower in RA/collagen than in renal transplant recipients.

### 3.4. Outcomes

Four patients (20%) died in the hospital, all of whom had received tracheal intubation. Three had RA, with two of the three having interstitial lung disease (ILD). These two patients were initially weaned from NPPV on days 4 and 7, but ILD worsened on days 6 and 8, respectively. NPPV was restarted, but tracheal intubation was performed on days 10 and 21. One RA patient was intubated due to the development of pneumothorax on day 11. All three patients showed no improvement of oxygenation after intubation and died. The remaining nonsurvivor had nephrotic syndrome and liver cirrhosis (Child-Pugh grade C). On admission, the patient presented acute kidney injury. She was intubated on day 2 and died of liver failure.

Sixteen patients (80%) survived, including all seven renal transplant recipients. One RA patient with ILD survived without acute exacerbation. Their duration of MV and length of hospitalization were 10.0 (54.0, 13.5) days and 24.0 (19.0, 40.5) days, respectively.

### 3.5. Multivariate Analysis of Prognostic Factors for 90-Day Mortality

Analysis between survivors and nonsurvivors was shown in Table 3. Nonsurvivors were older and had lower serum albumin levels at baseline compared with survivors. Multivariate analysis demonstrated that the incidence of ILD as an underlining disease [odds ratio (OR) 15; 95% CI 1.01–438.0; *p* = 0.0488] and serum KL-6 [OR 1.0025; 95% CI 1.000504–1.000667; *p* = 0.0052] were independently associated with 90-day mortality. The need for tracheal intubation, that is, NPPV failure, and PEEP levels in the first five days were not associated with death.

## 4. Discussion

In this study, we found lower mortality rate in mechanically ventilated patients with PCP compared with those reported in previous studies. Also, our study showed that PEEP during the first five days of MV was not associated with 90-day mortality, unlike a previous report [6]. Patients with RA who had ILD showed higher mortality. KL-6 at baseline was greater in nonsurvivors than in survivors. Only one patient (5%) developed pneumothorax during MV.

Previous studies have reported that the survival rate of PCP patients who required MV was quite low. For example, mortality rates were reported to be 76% in intubated patients and 91% in patients switched from NPPV to tracheal intubation [7]. Similarly, another study found that 3 of 4 intubated RA patients died, whereas all 12 patients without RA were intubated but survived after MV, and the mortality of RA patients was significantly low compared with non-RA patients [14]. Three of 4 nonsurvivors in RA had existing pulmonary fibrosis and died of subsequent exacerbation of interstitial pneumonia after resolution of the PCP. Of 21 HIV-negative PCP patients who required NPPV, 15 (70%) were switched to tracheal intubation, with 13 of the latter (87%) dying after MV [12]. We found, however, that the mortality rate in intubated patients was 36%, lower than those in previous studies, despite similar intubation rate (60%).

The causes for the different mortality rate are not confirmed from the study. One possibility is the different MV strategy and complications. First, the incidence of pneumothorax in previous reports ranged from 13 to 61% [6, 8, 10, 12, 15, 16]. The morality rate of mechanically ventilated PCP patients with pneumothorax ranged from 38 to 100% [4, 5, 8, 12], and development of pneumothorax appeared as one of the predictors for poor prognosis in PCP patients with [7] or without HIV [8]. In a previous study, NPPV avoided tracheal intubation in 67% of patients and decreased the incidence of pneumothorax from 37.5% to 8.3% [10]. In our ICU, NPPV is a routine initial support for all patients with acute hypoxemic respiratory failure and may explain the low incidence of pneumothorax (5%). Lower mean airway pressure associated with CPAP and less ventilator dyssynchrony with CPAP and APRV may also decrease pneumothorax. In addition, it is reported that 71% of NPPV failed in HIV-negative patients [12]. Delayed intubation could contribute to higher mortality [7]. Studies have shown that the mortality of intubated patients ranged from 59% to 91% [5–8, 10, 17, 18]. We also intubated earlier than the previous studies [5], that is, before patients showed respiratory muscle fatigue, and that probably contributed to the lower mortality rate (36%) in our study. The result that intubation had no association with death supports our hypothesis. Second, PEEP plays an important role in treating PCP, which often decreases lung volume [19, 20]. We titrated PEEP to find the appropriate and safe level in each patient. The average was 10.5 to 11.4 cmH_2_O and no substantial adverse effects were observed. Although it is difficult to compare with previous studies, higher PEEP to maintain lung volume and decrease transpulmonary pressure might contribute to better outcome. Patient characteristics may also contribute to differences in mortality. Most patients in our study regularly visited our hospital clinics for the treatment of their baseline diseases. This may be responsible for earlier start of treatment (3.5 days) compared to the previous study (8.4 days) [6], reducing mortality rate.

Serum concentrations of 1,3-*β*-D-glucan, LDH, KL-6, and SP-D increased at ICU admission, as previously reported [21]. However, in our study 1,3-*β*-D-glucan in 3 of the 9 patients with RA was lower than 31.1 pg/mL, the cutoff proposed for a diagnosis of PCP [21]. Lower 1,3-*β*-D-glucan level in patients with RA was observed in the previous report [14]. PCP was still diagnosed and treated in these three patients despite the low 1,3-*β*-D-glucan levels preventing the progression of the disease. However, it is necessary to evaluate the measured values in consideration of the underlying disease.

ILD is a risk factor for PCP in patients with RA [22] and their prognosis worsened when ILD was exacerbated during treatment with biologics [23]. KL-6, a biomarker for interstitial inflammation in the lungs, at the onset of respiratory failure was significantly higher in nonsurvivors than in survivors. Therefore, KL-6 may be a potential predictor for the prognosis, especially in patients with RA and coexisting ILD. A previous study [24] reported higher levels of 1,3-*β*-D-glucan and KL-6 in PCP patients with HIV, but neither was associated with the mortality rate, probably because of the different underlying diseases and lower rate of MV. Higher levels of KL-6 and SP-D in our study indicate that the extent of epithelial injury from the interaction between underlying disease and MV may determine the mortality in patients with PCP presenting with acute respiratory failure.

This study has potential limitations. This is a retrospective case series study performed in a single center. The number of patients included was small. This study was not designed to compare between CPAP and mandatory ventilation. The mortality rate of renal transplant recipients has been decreasing [25] and the patient population of our study might affect the lower mortality. Despite these limitations, the mortality in our results was low and suggests a potential for improved prognosis.

## 5. Conclusions

We found MV strategies to prevent pneumothorax including liberal use of noninvasive ventilation, and PEEP titration and disuse of mandatory ventilation may improve mortality in this setting. Underlying disease of interstitial lung disease was a risk factor and KL-6 may be a useful predictor associated with mortality in patients with RA. These findings will need to be validated in larger studies.

## Figures and Tables

**Table 1:** Demographic characteristics of subjects.

Case	Age	Underlying disease	PED (mg)	MTX (mg)	APACHE II	PaO_2_/FIO_2_ (mmHg)	PaCO_2_ (mmHg)	mPS (mg)	PEEP (cmH_2_O)			DON (days)	DOI (days)	DOV (days)	LOH (days)	Outcome
									Day 1	Day 3	Day 5					
1	66	RT	5	0	21	131	29.2	80	8	10	8	1	5	5	15	Survived
2	52	RT	10	0	20	106	25.9	16	25	20	18	1	13	13	24	Survived
3	66	RT	5	0	23	277	20.6	80	5	5	—	3	0	3	14	Survived
4	50	RT	5	0	16	104	29.8	80	12	18	12	1	9	10	54	Survived
5	56	RT	10	0	15	154	27.0	80	7	12	10	14	0	14	27	Survived
6	55	RT	5	0	12	166	21.8	80	10	10	14	11	10	20	102	Survived
7	47	RT	6.4	0	11	315	23.1	80	7	5	—	3	0	3	23	Survived
8	79	RA, ILD	5	10	16	150	20.5	500	6	7	7	5	0	5	56	Survived
9	69	RA	0	2.5	15	162	27.1	500	8	8	—	4	0	4	30	Survived
10	74	RA, ILD	6	8	19	108	34.5	500	8	10	10	11	31	42	42	Died
11	65	RA	0	10	21	113	29.1	500	12	10	10	10	0	10	47	Survived
12	79	RA	8	4	23	202	24.8	500	10	8	8	1	34	34	37	Died
13	76	RA, ILD, asthma	3	16	18	177	28.1	1000	10	10	10	10	21	31	32	Died
14	66	RA	2	12	19	186	30.1	500	8	8	8	9	0	9	29	Survived
15	37	SLE, APS, MCTD	7.5	0	24	123	26.8	1000	10	15	13	1	0	6	27	Survived
16	47	SLE, APS	14	0	21	262	26.6	500	8	10	8	6	0	6	24	Survived
17	76	NS, LC, hepatitis	15	0	29	95	22.4	80	10	22	20	1	10	11	105	Died
18	57	Stevens-Johnson Synd.	65	0	22	227	29.5	80	20	16	15	5	66	71	178	Survived
19	41	Ulcerative Colitis	25	0	18	140	37.0	80	15	14	14	0	12	12	61	Survived
20	86	Malignant lymphoma	45	0	18	125	36.9	80	10	10	8	0	5	5	80	Survived

RT: renal transplantation; RA: rheumatoid arthritis; ILD: interstitial lung disease; SLE: Systemic Lupus Erythematosus; APS: anti-phospholipid antibody syndrome; MCTD: Mixed Connective Tissue Disease; NS: nephrotic syndrome; LC: liver cirrhosis; PED: prednisolone-equivalent dose; MTX: methotrexate; APACHE: acute physiologic and chronic health evaluation; mPS: methylprednisolone; PEEP: positive end-expiratory pressure; DON: duration of NPPV; DOI: duration of intubation; DOV: duration of ventilation; LOH: length of hospitalization.

**Table 2:** Serum 1,3-*β*-D-glucan, LDH, KL-6, and SP-D levels at ICU admission in all patients and major subgroups. Data are presented as medians (interquartile range). RA: rheumatoid arthritis; LDH: lactate dehydrogenase; SP-D: surfactant protein-D.

	Total (*n* = 20)	RA/collagen (*n* = 9)	Renal transplant (*n* = 7)	*p* value
1,3-*β*-D-Glucan (pg/mL)	256.1 (64.4, 993.9)	71.4 (32.9, 206.3)	2600.0 (1121.5, 2880.0)	0.0043
LDH (IU/L)	468.5 (373.8, 588.5)	405.5 (338.0, 548.5)	498.0 (371.5, 539.0)	0.916
KL-6 (U/mL)	650.5 (406.3, 1391.3)	409.0 (383.0, 1390.0)	565.0 (475.0, 983.0)	0.608
SP-D (ng/mL)	193.0 (106.0, 445.0)	155.0 (95.2, 403.5)	193.0 (133.0, 216.0)	1.000

**Table 3:** Comparison between nonsurvivors and survivors. Data are presented as patient number (%) and mean (SD) or medians (interquartile range) as appropriate.

	Nonsurvivors (*n* = 4)	Survivors (*n* = 16)	*p* value
Age, year, mean (SD)	76.2 (2.1)	58.7 (13.3)	0.0196
Male, number (%)	0 (0%)	8 (50%)	0.068
APACHE II, mean (SD)	22.2 (5.0)	18.2 (3.8)	0.093
SAPS II, mean (SD)	40.0 (9.8)	36.7 (7.8)	0.486
Underlying diseases, number (%)			
Renal transplantation	0 (0%)	7 (43.7%)	0.101
Rheumatoid Arthritis	3 (75%)	4 (25%)	0.061
Interstitial lung disease	2 (50%)	1 (6.2%)	0.028
PED (mg), median (range)	7.0 (5.3, 9.8)	6.4 (5.0, 12.0)	0.528
Methotrexate use (mg), median (range)	6.0 (3.0, 10.0)	0.0 (0.0, 0.6)	0.088
Duration before treat (days), median (range)	1.5 (0.8, 4.0)	4.0 (1.8, 7.0)	0.629
Duration before ARF (days), median (range)	1.5 (0.8, 4.0)	4.0 (2.0, 7.0)	0.565
WBC (/*µ*L), mean (SD)	7992.5 (1734.4)	9862.5 (3523.5)	0.323
Albumin (g/dL), mean (SD)	2.3 (0.5)	2.9 (0.4)	0.027
*β*-D-Glucan (pg/mL), mean (SD)	67.7 (70.6)	1158.9 (1448.2)	0.157
LDH (U/L), mean (SD)	424.2 (59.6)	511.3 (211.5)	0.434
KL-6 (U/mL), mean (SD)	2700.3 (1673.1)	738.7 (566.6)	0.002
SP-D (ng/mL), mean (SD)	1433.3 (1968.3)	220.3 (168.5)	0.042
Worst P/F (mmHg), mean (SD)	145.5 (52.2)	171.3 (65.1)	0.475
PaCO_2_ on admission (mmHg), mean (SD)	27.5 (5.2)	27.6 (4.8)	0.968
Tracheal intubation, number (%)	4 (100%)	8 (44%)	0.245
Mechanical ventilation			
PEEP day 1 (cmH_2_O), median (range)	10.0 (9.5, 10.0)	9.0 (7.8, 12.0)	0.668
PEEP day 3 (cmH_2_O), median (range)	10.0 (9.5, 13.0)	10.0 (8.0, 14.3)	0.615
PEEP day 5 (cmH_2_O), median (range)	10.0 (9.5, 12.5)	10.0 (8.0, 14.0)	0.711
NPPV duration, days, median (range)	5.5 (1.0, 10.3)	3.5 (1.0, 6.8)	0.663
Days of intubation, days, median (range)	26.0 (18.3, 31.8)	2.5 (0.0, 9.3)	0.077
Days of ventilation, days, median (range)	32.5 (26.0, 36.0)	7.5 (5.0, 12.3)	0.068
Pulse therapy of methylprednisolone, number (%)	3 (75.0%)	6 (37.5%)	0.177
Length of hospitalization (days), median (range)	39.5 (35.8, 57.8)	29.5 (24.0, 57.3)	0.844

*Definition of Abbreviations*. APACHE, acute physiological and chronic health evaluation, SAPS; simplified acute physiological score, PED; prednisolone-equivalent dose, ARF; acute respiratory failure, WBC; white blood cell, LDH; lactate dehydrogenase, SP-D; surfactant protein-D, P/F; PaO_2_/FIO_2_, PEEP; positive end-expiratory pressure, NPPV; noninvasive positive pressure ventilation.
